# Conservative vs. surgical management of acute appendicitis in children with acute leukemia: a single-center retrospective case series

**DOI:** 10.3389/fped.2025.1722697

**Published:** 2026-01-12

**Authors:** Chuanyang Liu, Meng Shi, Jinhua Jia, Hongzhen Liu, Meng Kong

**Affiliations:** 1 Department of Pediatric Surgery, Children’s Hospital Affiliated to Shandong University, Jinan, China; 2 Department of Pediatric Surgery, Jinan Children’s Hospital, Jinan, China; 3 Department of Gastroenterology, Children’s Hospital Affiliated to Shandong University, Jinan, China; 4 Department of Gastroenterology, Jinan Children’s Hospital, Jinan, China

**Keywords:** acute appendicitis in children, acute leukemia, multidisciplinary collaboration, perioperative management, transumbilical single-site double-port laparoscopic appendectomy

## Abstract

**Objective:**

To explore the diagnostic challenges, individualized treatment strategies, and prognostic outcomes of acute appendicitis in children with acute leukemia and to provide practical evidence and clinical guidance for establishing a standardized multidisciplinary collaborative diagnosis and treatment protocol.

**Methods:**

This single-center retrospective case series analyzed the clinical data of children with acute leukemia complicated by acute appendicitis admitted between December 2020 and December 2024. All children were evaluated by a multidisciplinary team (MDT). Individualized treatment plans, including either conservative management or surgical appendectomy, were formulated based on MDT assessment. Clinical data were analyzed retrospectively to summarize diagnostic and therapeutic experience.

**Results:**

Among the 10 children in this study, appendicitis onset occurred during induction (*n* = 5), consolidation (*n* = 2), or maintenance (*n* = 3) chemotherapy. Seven children with uncomplicated appendicitis were successfully treated conservatively with anti-infective and hematopoietic support. The remaining three children, presenting with fecalith impaction or perforation, underwent emergency transumbilical single-site double-port laparoscopic appendectomy (TSSDPLA); pathology confirmed suppurative or gangrenous appendicitis.

**Conclusion:**

This small case series highlights that managing acute appendicitis in leukemic children requires individualized MDT-guided strategies. Conservative treatment was effective for uncomplicated cases, while TSSDPLA provided a feasible minimally invasive option for complications. Early resumption of chemotherapy was prioritized. All patients achieved infection control and continued leukemia therapy without mortality. These findings offer a preliminary management framework, pending validation in larger studies.

## 1 Background

Acute appendicitis is the most common surgical cause of abdominal pain in the pediatric emergency setting (1). However, its presentation in immunocompromised children, particularly those with acute leukemia, is often atypical due to chemotherapy-induced neutropenia and intestinal mucosal damage, which can mask the systemic inflammatory response (2–4). This leads to a significant diagnostic dilemma, as symptoms overlap with common chemotherapy side effects and severe pathology may develop with minimal abdominal signs (5). Persistent management challenges include the interpretation of atypical abdominal signs in neutropenia, controversy over perioperative platelet transfusion thresholds, and the lack of standardized timing for integrating anti-infective therapy with chemotherapy cycles (6).

Minimally invasive strategies like transumbilical single-site double-port laparoscopic appendectomy (TSSDPLA) may offer advantages in this context by potentially reducing incision-related complications and postoperative pain, which are critical considerations for immunocompromised patients needing timely chemotherapy resumption (5).

This study aimed to address these challenges by summarizing our single-center experience in managing acute appendicitis in children with acute leukemia. We evaluated a multidisciplinary team (MDT) approach, explored dynamic trends in inflammatory markers (CRP/PCT), and assessed outcomes of individualized treatment pathways, including conservative management and TSSDPLA.

## 2 Materials and methods

### 2.1 General information

This single-center retrospective case series analyzed the clinical data of children with acute leukemia complicated by acute appendicitis who were admitted to our hospital between December 2020 and December 2024.

#### 2.1.1 Inclusion criteria

1) Age ≤ 18 years; 2) Definitive diagnosis of acute leukemia confirmed by bone marrow puncture; 3) Diagnosis of acute appendicitis based on clinical and imaging findings during a chemotherapy cycle for leukemia.

#### 2.1.2 Exclusion criteria

1) Incomplete clinical data; 2) Onset of appendicitis while not undergoing active chemotherapy.

**Figure 1:**
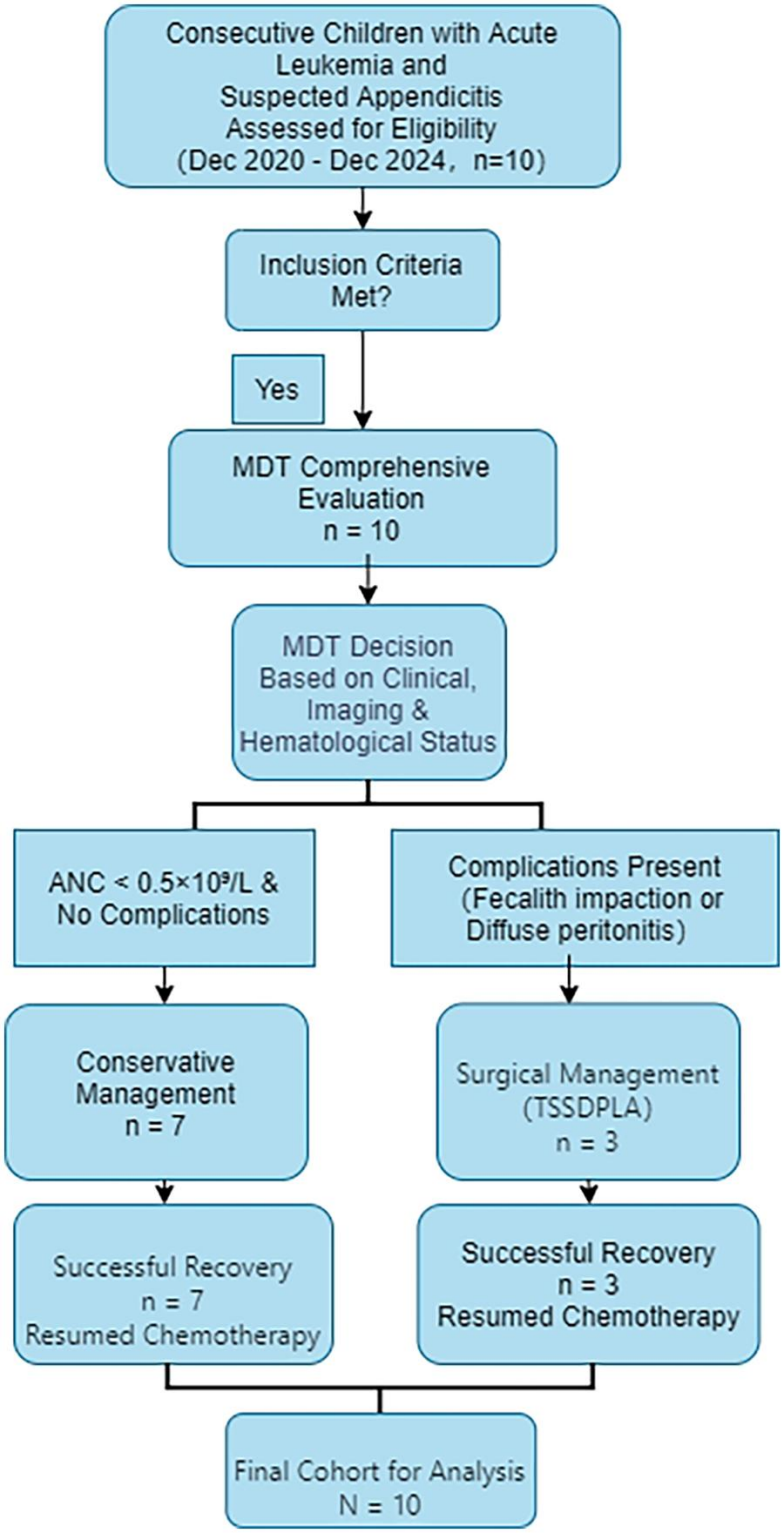
Flowchart of patient selection and management pathways.

All consecutive children with acute leukemia and suspected appendicitis who were assessed during the study period met the inclusion criteria and were included in the analysis. The flow of patient selection and management is summarized in Figure 1.

### 2.2 Diagnostic assessment

Clinical and laboratory evaluation: A detailed history was obtained, focusing on abdominal pain (location, migration, duration), fever (axillary temperature ≥38.0 °C), vomiting, and diarrhea. Physical examination assessed abdominal signs, including tenderness at McBurney's point, rebound tenderness, and guarding. Venous blood samples were collected at presentation for complete blood count with differential (white blood cell count, absolute neutrophil count, and platelet count) and inflammatory markers. C-reactive protein (CRP) was measured by immunoturbidimetry (normal <5 mg/L), and procalcitonin (PCT) was quantified using an electrochemiluminescence immunoassay (normal <0.05 ng/mL).

#### 2.2.1 Imaging studies

Bedside abdominal ultrasonography, performed by experienced pediatric radiologists, served as the first-line imaging modality. Sonographic diagnostic criteria for acute appendicitis included an outer appendiceal diameter >6 mm, non-compressibility, wall thickening (>2 mm), hyperemia on color Doppler, and/or the presence of periappendiceal fluid or abscess. For cases with equivocal ultrasound findings or persistent high clinical suspicion despite a negative scan, contrast-enhanced computed tomography (CT) of the abdomen and pelvis was obtained. CT criteria included appendiceal diameter >6 mm with wall enhancement, periappendiceal fat stranding, appendicolith, or extraluminal air.

#### 2.2.2 Final diagnosis

The definitive diagnosis of acute appendicitis and its complications (e.g., localized vs. diffuse peritonitis) was established through consensus by the multidisciplinary team (MDT), integrating all clinical, laboratory, and imaging data.

### 2.3 Treatment methods

After the diagnosis of acute appendicitis complicating acute leukemia, all the children in this study were comprehensively evaluated by a MDT, which included specialists from hematology, pediatric surgery, radiology, anesthesiology, and clinical pharmacy. Individualized treatment plans were jointly developed on the basis of the children's clinical manifestations, imaging findings, and hematological indicators. The management strategy for each patient included systematic anti-infective therapy, coagulation support therapy, and chemotherapy connection management.

#### 2.3.1 Conservative treatment

For children who met the indications for non-surgical treatment (no perforation, no diffuse peritonitis, and stable hemodynamics), a systematic and individualized combined conservative treatment plan (integrating drug and non-drug interventions) was adopted, covering four core modules: anti-infective therapy, immune function support, nutritional support therapy, and dynamic condition monitoring. The specific plan is as follows:

##### 2.3.1.1 Anti-infective therapy

After blood and peritoneal fluid samples were collected for pathogenic culture, broad-spectrum antibiotics were empirically administered immediately to cover common gram-negative bacteria and anaerobic bacteria. The regimen used in our center was piperacillin-tazobactam or cefoperazone-sulbactam combined with metronidazole or ornidazole. For children with a history of β-lactam antibiotic allergy or high infection risk, meropenem was used instead. For children with long-term neutropenia (ANC < 0.5 × 10^9^/L for more than 7 consecutive days) or those who received broad-spectrum antibiotics for more than 5 days, caspofungin or micafungin was added for antifungal therapy to prevent fungal infection.

##### 2.3.1.2 Immune function support

Children received subcutaneous injection of G-CSF at a dosage of 5 μg/kg/day. The ANC of the children was dynamically monitored, and the injection was stopped after ANC was stably maintained above 1.0 × 10^9^/L.

##### 2.3.1.3 Nutritional support therapy

A stepwise progressive nutritional support plan was adopted according to the children's intestinal tolerance, with enteral nutrition given priority. When children have contraindications to enteral nutrition (e.g., severe vomiting, intestinal obstruction) or insufficient enteral nutrition intake (unable to meet more than 50% of daily energy requirements), total parenteral nutrition is initiated, combined with glutamine to protect intestinal mucosal barrier function. After the children's condition improved, the transition to enteral nutrition was made as early as possible.

##### 2.3.1.4 Monitoring and adjustment

Abdominal signs (evaluated by the modified Alvarado scoring system), inflammatory indicators (CRP, PCT), and imaging changes in the children were comprehensively assessed every 12–24 h. If the child's condition did not respond well within 48–72 h of treatment (manifested as persistent fever, aggravated abdominal pain, or continuous elevation of inflammatory indicators), the anti-infective regimen was adjusted in a timely manner, and occult infection foci were screened by imaging.

**The efficacy evaluation criteria (all conditions must be met simultaneously)**:

Abdominal pain basically disappears (for children ≤6 years old or those unable to communicate effectively, as evaluated by the FLACC pain scale with a score ≤2; for children > 6 years old, evaluated by the Wong-Baker pain scale with a score ≤2);
Body temperature returns to normal and persists for ≥ 24–48 h;
The child can tolerate oral feeding normally;
Ultrasonography reexamination reveals that the appendiceal diameter is reduced by ≥ 30% compared with that before treatment, and periappendiceal effusion or abscess is significantly absorbed;
CRP and PCT significantly decreased, with CRP decreasing by > 50% compared with the peak value (or absolute value <10 mg/L) and PCT <0.5 ng/mL;
No intravenous infusion support is needed, the child can leave bed and move independently, and the mental state and appetite return to normal.

#### 2.3.2 Surgical treatment

All surgical interventions were performed using the transumbilical single-site double-port laparoscopic appendectomy (TSSDPLA) technique. The procedure emphasized meticulous techniques and stringent perioperative hematological management to mitigate risks in this immunocompromised cohort.

**Figure 2:**
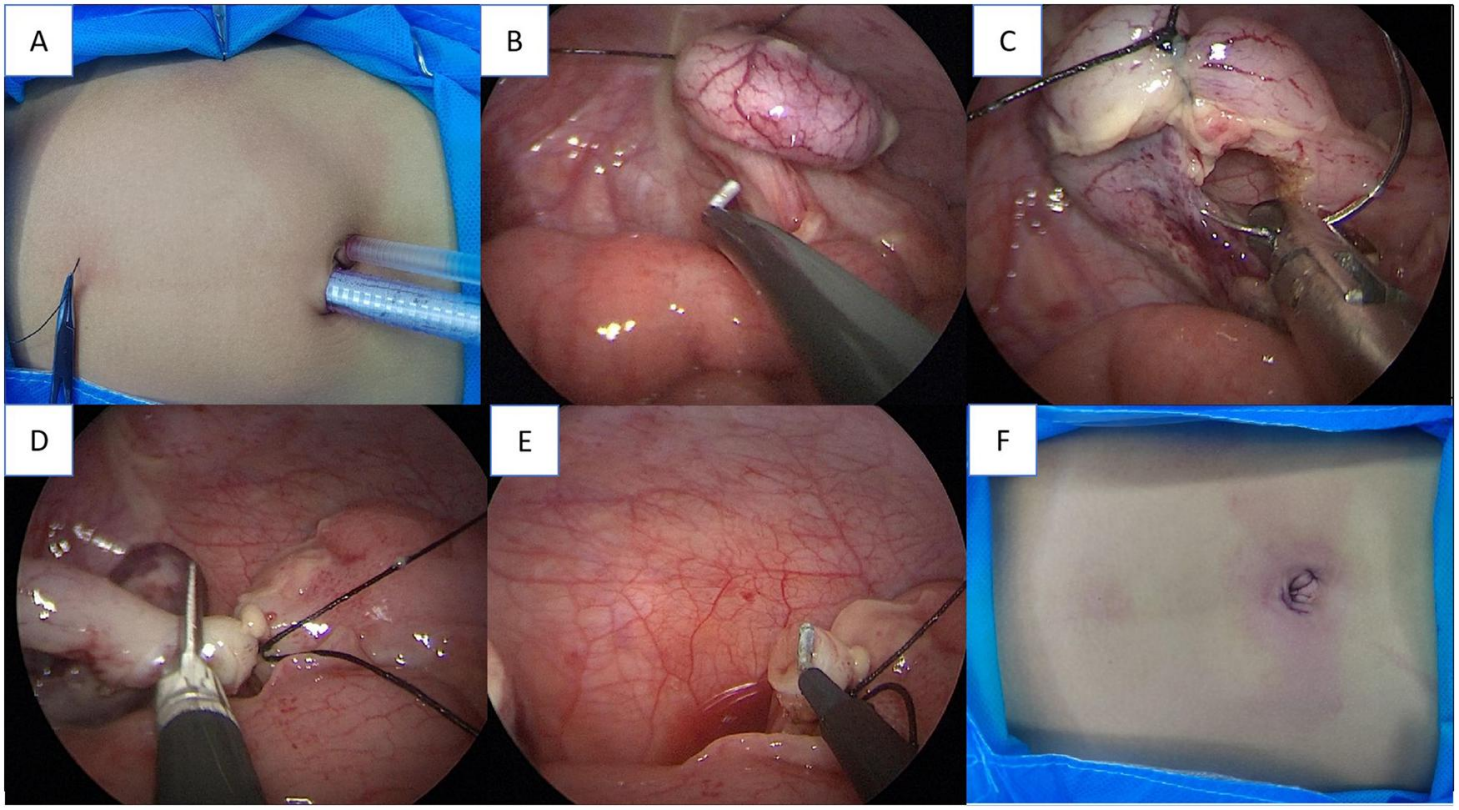
Key steps of transumbilical single-site double-port laparoscopic appendectomy (TSSDPLA). Placement of trocars and the traction sutures. **(B)** *In situ* electrocoagulation of the appendicular artery and mesentery. **(C)** Percutaneous suspension of the appendix using a seromuscular suture. **(D)** Transection of the appendix. **(E)** Closure of the appendiceal stump. **(F)** Final appearance of the umbilical wound after closure.

The key technical points included the following: establishment of operative access via 5-mm and 10-mm trocars placed around the umbilical ring (Figure 2A); exposure and traction of the appendix via a percutaneous silk suture suspension technique (Figure 2B); secure transection of the mesentery using a combined “bipolar electrocoagulation-ligation” method (Figure 2C); and ligation and transection of the appendiceal base (Figure 2D).

Perioperative hematological management was performed as follow: platelet transfusion was administered preoperatively to maintain a count >80 × 10^9^/L; fibrinogen levels were maintained above 2.0 g/L intraoperatively; and packed red blood cells were transfused immediately if the hematocrit fell below 25%. Early ambulation was encouraged postoperatively. As part of the protocol, the MDT conducted a joint evaluation to resume leukemia chemotherapy at the earliest feasible time, continuing G-CSF support if necessary to promote granulocyte recovery.

The comprehensive perioperative protocol, detailing technical and hematological measures for each surgical phase, is summarized below (see Table 2).

## 3 Results

The baseline characteristics, inflammatory markers, and treatment outcomes of the cohort, stratified by management strategy, are summarized in Table 1.

**Table 1:** Comparison of clinical characteristics and inflammatory trajectories between treatment groups.

Variable	Conservative Group (*n* = 7)	Surgical Group (*n* = 3)	Clinical Implication
Age, years	8 (4–12)	7 (7–13)	Comparable age distribution between groups.
WBC, ×10^9^/L	0.66 (0.33–2.06)	5.98 (3.65–8.48)	Higher baseline leukocyte count in the surgical group.
PLT, ×10^9^/L	55 (8–261)	95 (91–263)	Platelet counts showed wide variability in both groups.
ANC, ×10^9^/L	0.12 (0.01–1.42)	4.58 (3.22–4.85)	Profound neutropenia in the conservative group favored a non-surgical approach; preserved ANC in the surgical group permitted safer operative intervention.
Imaging Findings (MDT Decision Driver)	Localized inflammation, no complications	Fecalith impaction (*n* = 2), Perforation (*n* = 1)	Primary Indication for Surgery: The presence of complications (fecalith/perforation) was the key determinant for surgical management.
CRP at Diagnosis (Dx), mg/L	42.31 (28.8–233.8)	116.0 (114.0–144.6)	Markedly higher initial systemic inflammation in the surgical group.
CRP at Day 3 Post-Dx, mg/L	22.2 (11.6–70.2)	76.2 (66.8–84.5)	A rapid declining trend was evident in the conservative group.
CRP at Day 6 Post-Dx, mg/L	4.38 (2.42–13.79)	15.60 (4.42–17.50)	CRP levels approached normality in the conservative group; levels remained elevated in the surgical group, consistent with initial severity.
PCT at Diagnosis (Dx), ng/mL	0.54 (0.23–1.15)	0.72 (0.52–0.80)	Higher initial procalcitonin suggested greater severity of bacterial infection in the surgical group.
PCT at Day 3 Post-Dx, ng/mL	0.27 (0.18–0.65)	0.24 (0.22–0.35)	A downward trend was observed in both groups.
PCT at Day 6 Post-Dx, ng/mL	0.13 (0.11–0.21)	0.13 (0.12–0.14)	Both groups achieved similar PCT normalization
Treatment Duration, days	9 (7–13)	7	shorter hospitalization length was observed in the surgical group.

Data are presented as median (minimum–maximum). The time points for inflammatory markers are defined relative to the date of diagnosis (Dx).

ANC, absolute neutrophil count; WBC, white blood cell count; PLT, platelet count; CRP, C-reactive protein; PCT, procalcitonin.

### 3.1 Patient characteristics

The demographic and clinical characteristics of the cohort are summarized in Supplementary Table S1. The cohort included 10 children (6 males, 4 females) with a median age of 8 years (range: 4–13 years). Underlying leukemia subtypes were acute lymphoblastic leukemia (ALL, *n* = 5), acute myeloid leukemia (AML, *n* = 3), and T-lymphoblastic leukemia (T-LBL, *n* = 2). At the time of appendicitis onset, all children were on active chemotherapy, with 5 in the induction therapy phase, 2 in consolidation, and 3 in maintenance. The most common presenting symptoms were abdominal pain (9/10) and fever (7/10); vomiting and diarrhea were less frequent (each *n* = 2). Physical examination revealed fixed tenderness at McBurney's point in all children. Imaging (abdominal ultrasound or CT) confirmed appendiceal wall thickening (>6 mm) with associated effusion in all cases, leading to a diagnosis of localized peritonitis in eight children and diffuse peritonitis in two.

### 3.2 Non-surgical treatment group (*n* = 7)

**Figure 3:**
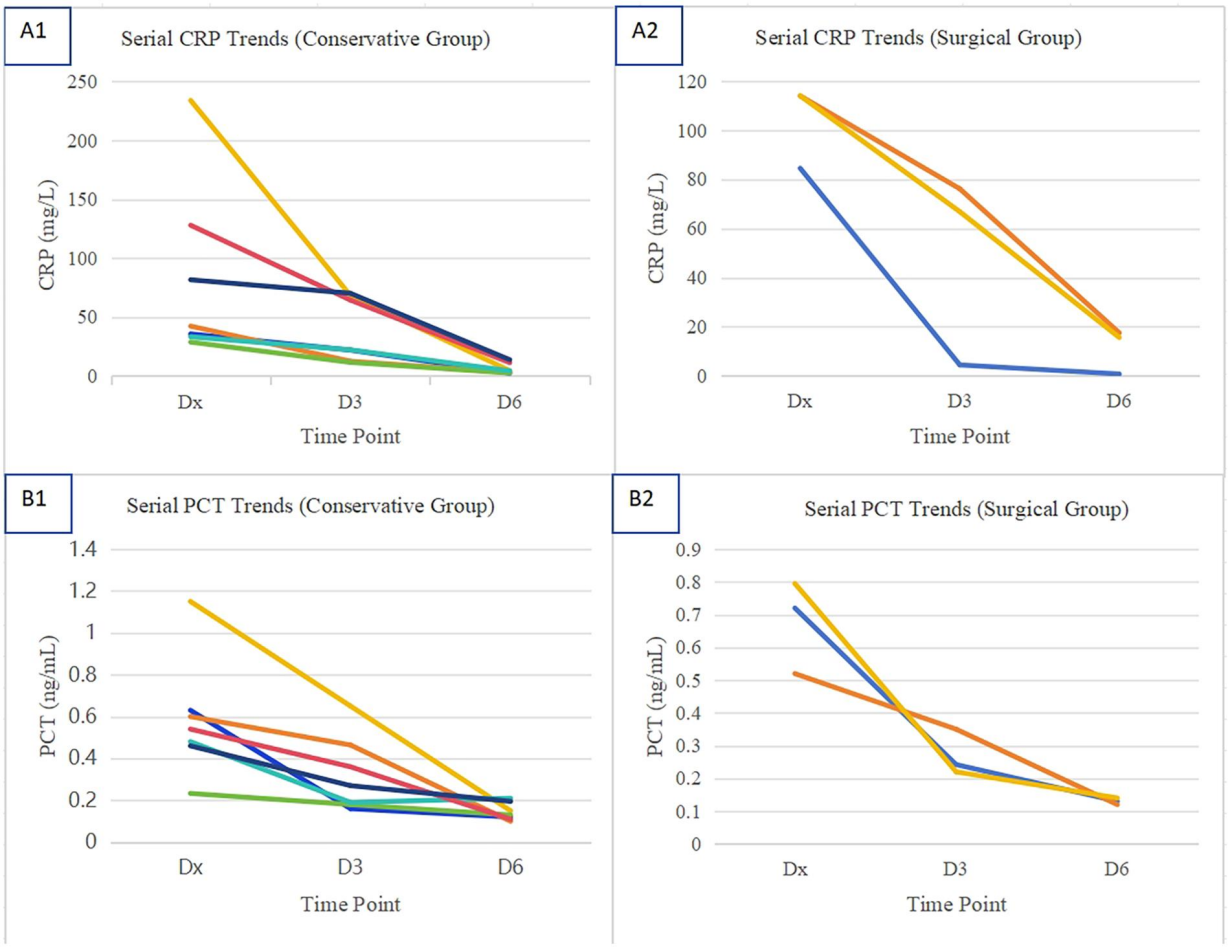
Longitudinal trends of inflammatory markers stratified by treatment group. **(A1)** Serial CRP levels in conservatively managed patients; **(A2)** Serial CRP levels in surgically managed patients; **(A3)** Serial PCT levels in conservatively managed patients; **(A4)** Serial PCT levels in surgically managed patients. CRP, C-reactive protein; PCT, procalcitonin.

All 7 children in this group received conservative treatment, with a treatment course of 7–13 days (median duration: 9.5 days). All children showed significant clinical improvement concomitant with the recovery of absolute neutrophil count (ANC) to >0.5 × 10^9^/L. This clinical recovery was supported by a consistent downward trend in serial inflammatory markers, as detailed in Table 1. After treatment, all the children entered the regular follow-up phase. The follow-up procedures included regular abdominal ultrasonography (to evaluate the appendix and abdominal cavity) and leukemia minimal residual disease monitoring (to evaluate the efficacy of leukemia treatment). In December 2024, the median follow-up duration of all the children was 12 months (range: 3–24 months). During the follow-up period, no recurrence of appendicitis was observed, and the leukemia of any of the children was effectively controlled. This clinical improvement was supported by a downward trend in inflammatory markers (Figure 3).

### 3.3 Surgical treatment group (*n* = 3)

**Figure 4:**
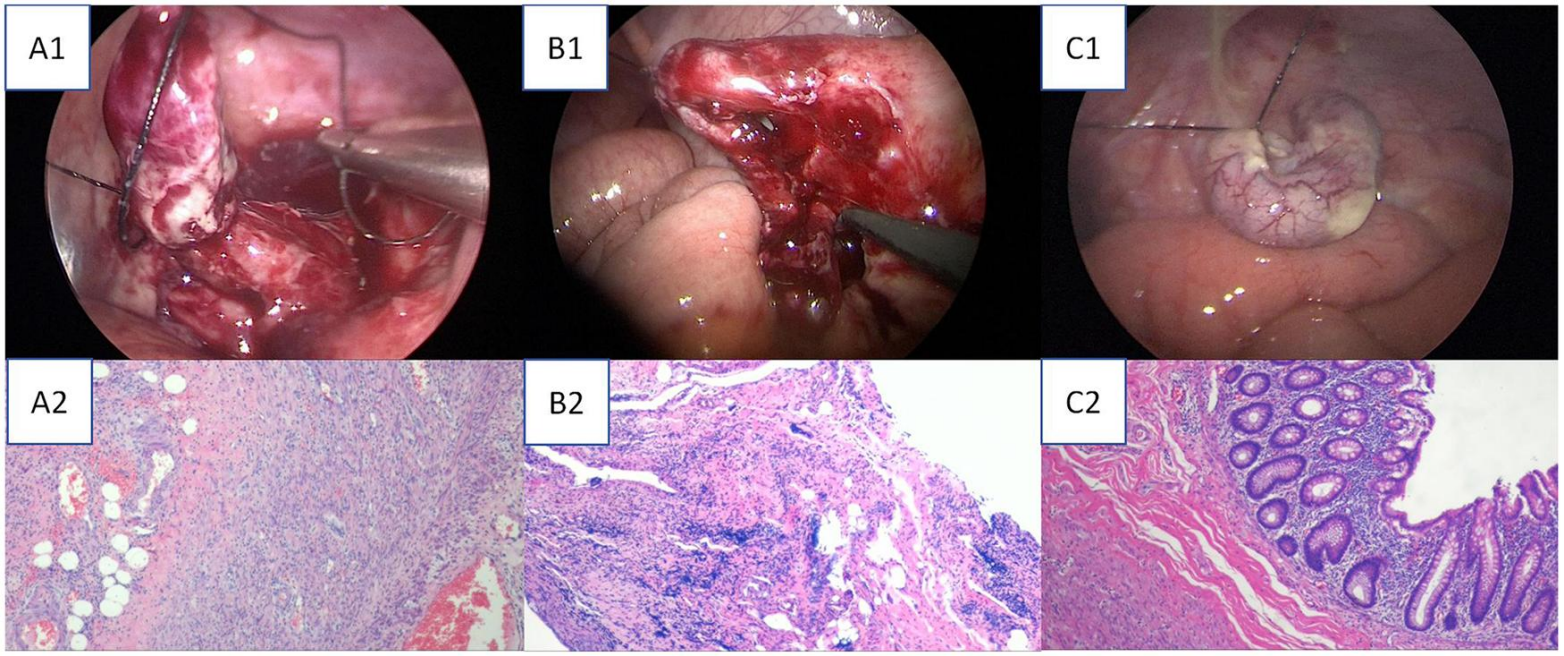
Intraoperative appendiceal photographs and corresponding postoperative pathological findings. **(A1)** Intraoperative view of a gangrenous and perforated appendix. **(A2)** Corresponding histopathology (H&E stain) confirming acute gangrenous appendicitis with transmural necrosis and perforation. **(B1)** Intraoperative view of an abscess-forming appendix. **(B2)** Corresponding histopathology (H&E stain) confirming periappendiceal abscess formation. **(C1)** Intraoperative view of a congested and edematous appendix, indicative of the acute suppurative process. **(C2)** Corresponding histopathology (H&E stain) confirming acute suppurative appendicitis with abundant neutrophilic infiltrate.

The median hospitalization duration of the 3 children in this group was 7 days, and the intestinal function recovery rate within 48 h after surgery was 100% (manifested as anal flatus and defecation, and tolerated a liquid diet). Postoperative pathological examination confirmed the preoperative and intraoperative findings, revealing acute suppurative appendicitis with fecalith impaction in two children and acute gangrenous appendicitis with perforation in one child. Representative histopathological images from the surgical cohort are provided in Figure 4. Pathogenic culture of the pus collected during surgery revealed extended-spectrum β-lactamase (ESBL)-negative Escherichia coli, and a drug susceptibility test revealed that the pathogen was sensitive to the empirically used antibiotics before surgery. No complications such as incision infection, peritoneal abscess, or bleeding occurred in any of the children during the perioperative period, and all the patients recovered well after surgery. All procedures were completed successfully according to the predefined TSSDPLA and perioperative management protocol (Table 2), without the need for conversion to open surgery.

**Table 2:** Key perioperative techniques and hematological guarantee measures for TSSDPLA in children with acute leukemia.

Surgical Phase	Key Technical Points	Hematological Guarantee Measures
Preoperative Preparation	Complete anaesthetic evaluation (ASA Grade Ⅱ-Ⅲ)	Preoperative platelet transfusion to achieve count >80 × 10^9^/L (including estimated intraoperative loss)
Access Establishment	Insert 5 mm and 10 mm Trocars respectively to the upper right and lower left of the umbilical ring to establish the operation channel (Figure 2A).	Maintain fibrinogen level >2.0 g/L intraoperatively
Appendiceal Management	The percutaneous suspension technique (using 2-0 with needle wire) was used to expose the appendix mesentery, and the mesentery was safely treated by the double method of “bipolar electrocoagulation-ligation” (Figures 2B,C).	Have adequate red blood cell suspension prepared; transfuse immediately if Hct <25% during surgery
Postoperative Management	Encourage early ambulation to promote intestinal function recovery.	After vital signs stabilize and infection is controlled, the MDT evaluates the patient to formulate an individualized plan for early resumption of leukemia chemotherapy; continue G-CSF support postoperatively if needed to promote granulocyte recovery.

TSSDPLA, transumbilical single-site double-port laparoscopic appendectomy; ASA, American Society of Anesthesiologists; MDT, multidisciplinary team; G-CSF, granulocyte colony-stimulating factor; Hct, hematocrit.

## 4 Discussion

The results of this study suggest that the clinical management of acute appendicitis in children with acute leukemia involves multiple disciplines including hematology, surgery, and radiology, presenting multidimensional complexity. From an epidemiological perspective, with the continuous optimization of modern intensive chemotherapy regimens, the 5-year survival rate of children with acute leukemia has increased to more than 90% (7); this improvement in survival has shifted the clinical focus to reducing treatment-related complications, including acute appendicitis. Therefore, the prevention and control of chemotherapy-related complications have become key issues in clinical diagnosis and treatment. Chemotherapeutic drugs (such as vincristine, doxorubicin, and methotrexate) have definite mucosal toxicity. This may directly damage the appendiceal mucosal barrier in children, thereby increasing the risk of appendicitis (8, 9). This is supported by a study demonstrating that chemotherapy-induced intestinal mucositis, quantified by biomarkers of enterocyte loss (citrulline) and mucosal inflammation (CCL20), significantly compromises gastrointestinal barrier integrity and increases the risk of systemic complications during induction therapy (10). In this small case series, we observed that half of the appendicitis cases (5/10) occurred during the leukemia induction remission phase, during which patients often have significant myelosuppression and delayed immune reconstitution. This finding is somewhat consistent with literature suggesting that “chemotherapy intensity is positively correlated with the risk of complications” (11).

In terms of diagnosis, the research team identified three core challenges: First, traditional inflammatory markers (e.g., WBC count) lose diagnostic value in children with myelosuppression. In our cohort, the average WBC count was only 2.5 × 10^9^/L (vs. typical elevation in acute appendicitis), risking misdiagnosis. A Tel Aviv Medical Center study on 14 AL children with AA further confirmed this finding—the median ANC was 0.15 × 10^9^/L at AA diagnosis, with most having persistent severe neutropenia, limiting marker utility (12). Second, glucocorticoids for leukemia may mask signs of peritoneal irritation signs. Only mild right lower abdominal tenderness was observed in 3 children here, with no typical signs such as muscle rigidity or rebound tenderness. This aligns with the CCCG-ALL-2015 study (13) and is further supported by immunocompromised patient research (14, 15); Third, gastrointestinal reactions caused by chemotherapeutic drugs (e.g., abdominal pain, nausea, vomiting) overlap with the symptoms of appendicitis, making distinguishing between the two conditions on the basis of symptoms alone difficult. On the basis of the above diagnostic challenges, we adopted a multi-modal diagnostic pathway in our practice: For children with persistent abdominal pain for more than 6 h, bedside ultrasonography should be performed immediately to evaluate the appendix. For children with unclear ultrasonic findings, enhanced CT examination should be further performed to effectively distinguish between appendiceal mucosal edema and fecalith impaction, thereby improving diagnostic accuracy. International guidelines on acute appendicitis (SAGES/EAES) specifically highlight that multimodal imaging (ultrasound as the first-line, supplemented by CT or MRI) is crucial for the diagnosis of special populations, which strongly supports our diagnostic approach (14).

The selection of treatment strategies in our practice was guided by a dynamic risk assessment model and adjusted in a timely manner according to the child's condition changes. For children without appendiceal perforation or peritoneal abscess, the intensive conservative treatment regimen adopted in this study (broad-spectrum antibiotic therapy + parenteral nutrition support + component blood transfusion therapy) achieved a high rate of success in this cohort. The successful implementation of this regimen involves two key steps: First, a combined antibiotic strategy covering gram-negative bacteria and anaerobic bacteria was adopted in the initial treatment phase to ensure timely control of the infection source and avoid infection spread. This is partially consistent with the perspective of the SAGES/EAES guidelines that nonoperative management can be considered for specific pediatric appendicitis cases; Second, in our management, the child's platelet level was maintained above 50 × 10^9^/L during treatment—a safety threshold that is widely recognized and applied in key areas of pediatric care, including oncology and hematopoietic cell transplantation (16).

Surgically, this study utilized TSSDPLA to treat acute appendicitis in children with acute leukemia. Compared with traditional three-port laparoscopy, TSSDPLA may offer several potential advantages for immunocompromised children: First, concentrating operating channels at the umbilicus may reduce the risk of incision infection; Second, fewer incisions can alleviate postoperative pain; Third, concealed scars improve cosmetic outcomes (17, 18). Notably, the use of specialized laparoscopic traction devices (e.g., the device developed by Geng et al.) can further optimize the exposure of the appendiceal mesentery during TSSDPLA, which is particularly valuable for children with narrow abdominal cavities and limited surgical space (17). Additionally, a cost-analysis study of laparoscopic appendectomy in integrated healthcare systems suggested that TSSDPLA may maintain controllable overall treatment costs compared to the conventional approach, primarily through reduced trocar utilization (5, 18). While our study did not conduct a formal cost-analysis, this potential benefit is worth noting for leukemic children who require long-term cancer care. Intraoperative pus culture in our study identified Escherichia coli as the main pathogen, which supports the use of third-generation cephalosporins combined with metronidazole as empirical anti-infective therapy, which is consistent with the pathogen distribution and antibiotic selection recommendations in acute appendicitis reviews (19).

The clinical implications of this study are threefold: First, establishing an MDT was essential in our center for developing individualized diagnosis and treatment pathways, as an MDT has been shown to reduce misdiagnosis and complication rates in pediatric hematology-oncology patients with abdominal diseases. Second, future research should focus on developing a perforation risk prediction model based on ultrasound or CT radiomics to achieve early risk stratification; Third, the potential of intestinal flora modulation in preventing chemotherapy-related mucosal damage deserves attention. A study revealed that probiotic supplementation can reduce intestinal mucosal inflammation in children with ALL, and recent research revealed that appendectomy-induced alterations in the gut microbiome composition may have long-term impacts on gastrointestinal health, which reminds us to consider microbiome protection when managing appendicitis in leukemic children with pre-existing intestinal barrier impairment (20).

## 5 Limitations

This study has several limitations. First, the small cohort of only 10 patients significantly limits generalizability and precludes robust statistical comparisons. Second, its single-center, retrospective design may introduce institutional bias. Third, the absence of a control group prevents definitive comparison of our strategies against other approaches. Finally, the lack of long-term oncologic outcomes means the impact of the appendicitis episode and any chemotherapy interruption on leukemia survival and relapse remains unclear.

## 6 Conclusion

In our experience, the management of acute appendicitis in children with acute leukemia benefited from a multidisciplinary collaborative system. In our small series, intensive conservative management was successful for non-perforated cases, while TSSDPLA proved a feasible minimally invasive surgical solution. Future efforts should focus on developing risk-prediction models and exploring gut microbiome modulation. This study provides a preliminary practical framework for addressing this challenging complication, which requires future validation.

## Data Availability

The raw data supporting the conclusions of this article will be made available by the authors, without undue reservation.
